# Engaging suicide prevention and firearm stakeholders in developing a workshop promoting secure firearm storage for suicide prevention

**DOI:** 10.1186/s40621-024-00511-7

**Published:** 2024-06-14

**Authors:** Hanna Christian, Dev Crasta, Garra Lloyd-Lester, Gala True, Marianne Goodman, Brett Bass, Kathryne Coric, Timothy Ruetten, Robert Lane, Gabriela Khazanov

**Affiliations:** 1 University of Pennsylvania Perelman School of Medicine, Philadelphia, PA USA; 2 University of Rochester Medical Center, Rochester, NY USA; 3 Suicide Prevention Center of New York, Menands, NY USA; 4 Veteran-Informed Safety Intervention and Outreach Network, New Orleans, LA USA; 5 VISN 2 Mental Illness, Research, Education and Clinical Center, Department of Veterans Affairs Medical Center, James J. Peters, Bronx, NY USA; 6 Icahn School of Medicine at Mount Sinai, New York, NY USA; 7 University of Washington School of Social Work, Seattle, WA USA; 8 Suicide Prevention Coalition of Erie County, VA Western New York Healthcare System, Buffalo, NY USA; 9 Jefferson County Community Services, Watertown, NY USA; 10 Corporal Michael J Crescenz VA Medical Center, Philadelphia, PA USA

**Keywords:** Firearm safety, Suicide prevention, Secure firearm storage, Community-based participatory research

## Abstract

**Background:**

In the US, over 50% of suicide deaths are by firearm injury. Studies have found that limiting access to firearms, including storing them temporarily outside of the home or locking and unloading them securely at home, helps prevent suicide. Family members and other loved ones are in a unique position to encourage secure firearm storage. This paper describes the development of a workshop to empower loved ones of individuals at risk for suicide to discuss secure firearm storage in New York State.

**Methods:**

Using a multistakeholder engagement framework, we partnered with New York State county-level suicide prevention coalitions, local firearms experts, and other stakeholders to develop a 90-min workshop addressing secure firearm storage for suicide prevention. Pilot workshops were co-facilitated by a suicide prevention coalition member and a local firearms expert. Feedback gathered via surveys from workshop attendees and interviews with workshop co-facilitators were used to revise workshop content and inform dissemination. Following pilot workshops, a 1-day training event was held for potential future facilitators, and survey data were collected to assess trainee experiences and interest in facilitating future workshops. Data analysis included rapid qualitative analysis of interviews and statistical analysis of survey responses about acceptability of workshop.

**Results:**

Four pilot workshops included a total of 23 attendees. Pilot workshop attendees endorsed willingness and confidence to discuss secure firearm storage with a family member or loved one. The training event included 42 attendees, of which 26 indicated interest in facilitating a workshop within the next year. Co-facilitators agreed on several key themes, including the importance of having a “trusted messenger” deliver the firearms portion of the workshop, keeping the conversation focused on firearm safety for suicide prevention, and developing interventions that reflect firearm owning community’s culture.

**Conclusions:**

Consistent with a public health approach to suicide prevention, this study leveraged a multistakeholder engagement framework to develop a community-based workshop empowering loved ones of individuals at risk for suicide to discuss secure firearm storage. The workshop will be disseminated across New York State. We noted positive and collaborative relationships across stakeholder groups, and willingness to facilitate the workshop among both suicide prevention and firearm stakeholders.

**Supplementary Information:**

The online version contains supplementary material available at 10.1186/s40621-024-00511-7.

## Background

In the United States, suicide is the 12th leading cause of death and the majority of suicides deaths are by firearm injury (53%) (2023 National Veteran Suicide Prevention Annual Report 2023; Centers for Disease Control and Prevention, National Center for Health Statistics 2023). Among firearm fatalities, suicide is the leading cause (54%), outnumbering fatalities due to homicide, legal intervention, and undetermined and unintentional injury (Multiple Cause of Death Data on CDC WONDER 2023). Firearm ownership and access to firearms have been consistently linked to an increased risk of death by suicide (Anestis and Houtsma 2018; Bond et al. 2023). As veterans have nearly twice the rate of personal firearm ownership (50% vs. 30% in the general population) they also have correspondingly higher rates of firearm suicide (i.e., 71% compared to 50% among the general population) (2023 National Veteran Suicide Prevention Annual Report 2023; Fischer et al. 2023; Schweitzer 2021). Secure storage of firearms and limiting access to firearms during periods of higher suicide risk can lower the risk of death by suicide (Shenassa et al. 2004). Although there are a range of secure storage measures, the most common definition of secure firearm storage includes storing a firearm locked, unloaded, and stored separate from the ammunition, with a recommendation to take further steps during periods of increased risk (e.g., temporary transfer to a loved one; storage out of the home) (Safe Storage of Firearms 2024). Unfortunately, approximately a third of veteran firearm owners store at least one firearm in their home loaded and unlocked, greatly increasing the risk of suicide by firearm (Simonetti et al. 2018a).

Discussing the risks of access to firearms and benefits of secure storage may lead individuals to make changes to their storage practices (Albright and Burge 2003; Barkin et al. 2008; Rowhani-Rahbar et al. 2016). Education on secure firearm storage options is also associated with greater likelihood of using locking devices to secure firearms (Bandel et al. 2023). Additionally, community-based interventions can improve secure storage among firearm owners (Simonetti et al. 2018b; Stuber et al. 2021). Studies of active duty servicemembers suggest family members and loved ones of firearm owners are in a unique position to encourage secure storage of firearms and implement secure storage solutions, as well as help individuals at risk for suicide recognize warning signs and seek treatment (Dempsey et al. 2019). Given that living in a home that has a firearm in it increases the risk of death by suicide for all household members, secure firearm storage may also benefit others aside from the firearm owner themselves, particularly if children, teens, or other individuals at higher risk for injury (e.g., those who use alcohol or substances, individuals with cognitive impairment) live in or visit the home (Pallin and Barnhorst 2021).

To date, most efforts to encourage conversations about secure firearm storage have been aimed at clinicians and health care providers (Pallin and Barnhorst 2021). Recently, there have also been efforts to promote lethal means safety training among firearm retailers and instructors, and provide lethal means training to the general public (Houtsma et al. 2023; Constans et al. 2023; Ellison et al. 2023). Research has shown that when designing firearm-related interventions, it is critical to engage firearm stakeholders (i.e., individuals with expertise in firearms such as firearm retailers or trainers, law enforcement and military personnel, or veterans), as firearm owners prefer messaging about secure storage that is aligned with their values and comes from individuals deemed trustworthy (Garverich et al. 2023; Marino et al. 2017; Pallin et al. 2019). Family members have also been endorsed by firearm owners as credible messengers to discuss secure firearm storage for suicide prevention (Bond et al. 2022; Anestis et al. 2021). This research highlights the importance of choosing the right messenger along with the right message (Houtsma et al. 2023; Boine et al. 2022; Ewell Foster et al. 2023). Research has also found that interventions are more effective when they are developed together with the community they are impacting (Siddiq et al. 2023).

Consistent with this emerging literature, the current study sought to develop and disseminate a workshop to empower family members and loved ones of firearm owning community members to talk about secure firearm storage for suicide prevention. The content of this workshop was inspired by “Worried About a Veteran” (WAV), a website that provides Veteran family members with information on warning signs of suicide and ways to encourage secure firearm storage (https://worriedaboutaveteran.org/). WAV was developed by the lethal Means Workgroup of the New York State Governor’s Challenge and based on feedback from interviews with family members of veterans who had a firearm suicide attempt or had ongoing suicidal ideation while currently owning a firearm.

The current project builds on the success of the WAV website to provide families with more in-depth training and practice discussing secure firearm storage in a workshop format, as well as build connections to others in the community with similar concerns. In contrast to WAV’s “indicated prevention” approach, focusing on individuals with concrete concerns about a high-risk veteran, the workshop described in this study is a “selective prevention” approach that is focused on loved ones of firearm owners broadly, regardless of that owner’s veteran status or whether the attendee is aware of any specific suicide risk signs at the time of their attendance.

For this project, we used an approach for engaging multiple stakeholder groups aligned with the Engagement Rubric developed by the Patient-Centered Outcomes Research Institute (PCORI) (Sheridan et al. 2017; Valentine et al. 2021). The PCORI engagement rubric is guided by and based on the following principles: reciprocal relationships, partnerships, co-learning, and transparency-honesty-trust. We adapted these guidelines for the firearm suicide prevention context using published firearm safety and suicide prevention practice guidelines, community participatory based research standards of practice, and peer-reviewed literature, along with critical feedback by a panel of stakeholder groups (e.g., firearm experts, subject matter experts in family processes and suicidology, and family members of veterans).

Here we provide a detailed description of our approach to engage multiple stakeholder groups in the development and dissemination of a workshop aimed to provide family members and loved ones with more in-depth training and practice discussing secure firearm storage and suicide prevention with concerned significant others.

## Methods

### Multistakeholder engagement approach

As noted above, we adopted a multistakeholder engagement approach, informed by the PCORI engagement rubric (Sheridan et al. 2017). Multistakeholder engaged research seeks to incorporate the perspectives, insights, and expertise of various stakeholders such as community members, researchers, practitioners, and representatives from relevant organizations (Valentine et al. 2021). This collaborative model goes beyond traditional research methods, emphasizing shared decision-making, co-creation of knowledge, and the development of interventions that are not only informed by academic expertise but also grounded in the lived experiences and priorities of the community. For this research, we sought our three stakeholder groups known to be integral and relevant to firearm owning individuals and suicide prevention. Throughout workshop development, the research team met with each stakeholder group several times to communicate changes to the workshop, promote transparency and trust between research team and stakeholders, and ensure input of all stakeholders. This multistakeholder engagement process was classified as workshop development was not considered research and was conducted with a waiver from the University of Pennsylvania Institutional Review Board.

### Stakeholders/partners and recruitment

Participants included three stakeholder groups: (1) five New York State county-level suicide prevention coalition (SPC) members (n = 6; one SPC was represented by two individuals due to scheduling conflicts), (2) subject matter experts in the fields of suicidology, firearm safety, and family processes (n = 8), local firearm experts (n = 7; e.g., trainers, retailers), and (3) family members of veterans (n = 6).

#### New York State county-level suicide prevention coalitions

We partnered with five New York State SPCs from counties with a high proportion of veteran residents (6% or above) compared to the New York State average (~ 4%), who represented diverse populations and both urban and rural areas. Each SPC had an effective track record of developing community education programs addressing a wide range of suicide prevention topics to a wide range of audiences (e.g., mental health advocacy in schools, tabling at gun shows, gatekeeper trainings in hospitals). SPCs were contacted by one of the authors (GLL), the coordinator of community and SPC initiatives at the Suicide Prevention Center of New York State, via email. All SPCs that were contacted agreed to participate.

#### Subject matter experts

The study team recruited subject matter experts by identifying and contacting individuals with relevant professional experience, including current and former collaborators on similar studies. Subject matter experts included researchers and clinicians with expertise in lethal means safety, Veterans’ health, stakeholder engagement, and family processes.

#### Firearm experts

Local firearm experts were recruited by New York State SPC members both through “cold approaches” (e.g., emailing gun shop owners) as well as identifying appropriate individuals through their professional and personal networks. Support for finding firearm experts (e.g., generating lists of potential experts) was provided by the research team upon request by SPC members. The final firearm experts identified included an owner of a gun shop, a registered firearm instructor, leaders of a community gun club/advocacy organization, and law enforcement officer.

#### Veteran family members

Veteran family members were identified through the two organizations focused on supporting family members of individuals at risk for suicide or those impacted by a family member’s death by suicide. Members of the organizations’ leadership teams contacted potentially interested family members, who were put in touch with the research team. Due to the sensitive nature of these meetings, they were not audio recorded.

### Pre-implementation stakeholder meetings

**Fig. 1:**
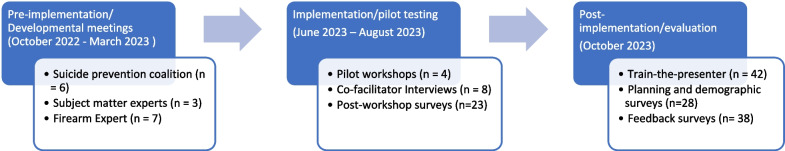
Workshop development by implementation phase

Pre-implementation stakeholder meetings were conducted virtually via videoconference. We met with each stakeholder group together (e.g., New York State SPCs, subject matter experts, and veteran family members) and individually with local firearm experts to better accommodate their schedules. Workshop materials and the facilitator guide were developed through review of relevant literature and discussion with the stakeholder groups. The workshop materials and facilitator guide were repeatedly presented for comment and review, with iterative revisions made after each meeting. Consent and permission to audio record were confirmed, and sessions lasted approximately 60 min. Meetings were structured to make efficient use of stakeholders’ time and to allow adequate space to gather input and facilitate group discussion. Field notes were taken during each meeting by a member of the research team (HPC). Firearm experts and veteran family members, who met outside of their professional scopes of work, were offered $50 for each meeting to compensate them for their time. Details on stakeholder engagement and research activities by implementation phase can be found in Fig. 1.

### Workshop feedback

Pilot workshops were evaluated through two separate strategies. All evaluation procedures for these post-workshop evaluations were reviewed and approved by the University of Pennsylvania Institutional Review Board.

#### Pilot workshop attendee surveys

At the end of every pilot workshop, attendees were asked to complete a one-page, anonymous survey that assessed pre- and post-workshop perspectives on secure firearm storage and suicide prevention [see supplement for full survey]. Workshop facilitators distributed surveys to attendees at the end of the workshop, and attendees received a small gift (i.e., LED pocket flashlight or $5 amazon.com gift card for attendees of the virtual workshop) for completing the survey.

#### Co-facilitator interviews

Semi-structured interviews were conducted separately with each co-facilitator within two weeks of delivering the pilot workshop by an interviewer (GKK) and trained research coordinator (HPC). The interview guide included the following domains: the makeup of the audience, experiences delivering the workshop, working with the co-facilitator, requests for changes to the facilitator guide and workshop materials, and plans to deliver the workshop in the future [see supplement for interview guide]. Consent and permission to audio record were confirmed, and interviews lasted 30–60 min. Participants were given the option to be compensated $50 for their time.

### Training-the-presenter event

Following pilot workshops and revision of materials based on attendee surveys and co-facilitator interviews, a 1-day ‘train-the-presenter’ event was held for new workshop presenters to disseminate the workshop across New York State. The event was advertised via a County Suicide Prevention Coalition listserv and social media. The event included a brief overview of the project, a presentation of the workshop by a SPC member and local firearm expert, group discussions on workshop content, networking activities, and a panel discussion with pilot workshop facilitators on best practices for delivering the workshop. Reimbursement was offered for travel and one night’s lodging for attendees.

#### Train-the-presenter event attendee surveys

In-person train-the-presenter event attendees were given the opportunity to complete two surveys: a one-page, anonymous feedback survey on workshop content, training activities and demographics, and a one-page, identified survey assessing attendee’s area of expertise and plans to facilitate the workshop within the next year.

### Data analysis

#### Stakeholder meetings

All interviews were recorded and transcribed verbatim by a trained research coordinator (HPC) assisted by an automatic transcription software (Liang et al. 2016). Analysis followed a rapid qualitative analysis approach (Hamilton 2020; Lewinski et al. 2021; Vindrola-Padros and Johnson 2020). One member of the research team (HPC) coded all stakeholder meeting transcripts using a summary template that corresponded with the domains of the meeting agenda. A second member of the research team (GKK) reviewed all coding, and the two coders discussed and resolved discrepancies that came up. This included a review of each stakeholder meeting transcript and corresponding field note and meeting agenda (Maietta et al. 2021).

#### Co-facilitator interviews

Co-facilitator interviews were also analyzed using a rapid qualitative analysis approach designed to produce immediate, actionable results (Hamilton 2020, 2013; Lewinski et al. 2021). During the interviews, a trained research coordinator (HPC) developed detailed summaries using a template that corresponded with the domains on the interview guide that was then reviewed by a second member of the research team (GKK; see “Supplementary Materials”). Using an iterative process, two members of the research team (HPC and GKK) compiled a feedback action plan based on the suggestions made during the interviews and identified salient themes related to workshop feasibility, acceptability, and implementation.

#### Pilot workshop and train-the-presenter attendee surveys

Surveys were developed by adapting questions from previous lethal means safety and suicide prevention training evaluations. We calculated percentages, means, and standard deviations to examine responses to close-ended questions. For open ended questions, two authors (HPC and GKK) collaboratively identified themes and categorized each response accordingly.

## Results

### Pre-pilot workshop meetings

Consistent with a multistakeholder engagement framework, we sought input on workshop components from each stakeholder group. We sought feedback from each stakeholder group separately to ensure that group members felt comfortable providing feedback and that their perspectives were heard (Table 1). We received input on every workshop component by subject matter experts with professional expertise in workshop content, the SPC members and local firearm experts who would deliver the workshop, and the family members that represented our target audience.

**Table 1:** Workshop development

Theme	Suicide prevention coalition	Subject matter experts	Firearm experts	Veteran family members	Post-workshop feedback from co-facilitators
Scope and frame of workshop	Workshop should focus on suicide prevention, through the lens of secure firearm storage	The workshop should encompass a broad scope, potentially concentrating on firearm safety overall rather than exclusively on suicide prevention	Firearm experts endorsed community-based firearm suicide prevention efforts	The importance of providing information to family members and loved ones of individuals at risk, particularly Veterans, was stressed	Frame and scope of workshop should align with coalition’s area of expertise, which is suicide prevention
Format and structure of workshop	Workshop initially a 1-h session with modules for specific populations. Workshop should include dyadic portion to elicit audience engagement	How to approach conversations around concerns for suicide as well as discussions on secure firearm storage	Recommended workshop should be delivered in person for demonstrating secure storage options	Not applicable	The workshop’s length increased to 90 min in a modular format. Recommendations for in-person delivery due to sensitivity of content but also endorsed virtual workshop delivery
Target audience	Workshop should target family members and loved ones of firearm owners	Workshop should either target firearm owners or loved ones of firearm owners. Content may not be best suited for those with service weapons	Workshop target audience should include all community members interested in firearm suicide prevention	The significance of reaching out to the family members and loved ones of Veterans, was emphasized	Workshop target audience includes all community members interested in firearm suicide prevention, with a focus on loved ones and family members of firearm owners. Co-facilitators identified a need to increase focus on community groups for identification of future attendees
Suitability of workshop content	Suicide prevention coalition members emphasized suicide prevention, acknowledged the difficulty of conversations, and included resources for self-help	Workshop content should align with firearm owner values and include direct language on suicide	Firearm experts emphasized collaborative, voluntary, and temporary limits in access to firearms during elevated suicide risk	Veteran family members offered input on scenarios for discussion and practice	Slides and handouts were revised and shortened based on co-facilitators' feedback
Process for co-facilitating	It is important to meet with firearm expert co-facilitator prior to delivering the workshop to discuss logistics of workshop and comfortability with content	Information about firearms should be delivered by a local firearms expert	Firearm experts emphasized the importance of using credible messengers to deliver relevant firearm information	Not applicable	Facilitator’s guide was revised to include additional guidance on engaging with co-facilitators

#### Suicide prevention coalition meetings

Five meetings were held with SPCs between October 2022 and March 2023. Attendance of meetings was high (total attendance rate = 88% of SPC members who committed to attending, n = 6). No SPC member missed more than one meeting. The meetings focused on the framing, scope, and content of the workshop, finding and collaborating with a firearm expert to co-facilitate the workshop, and identifying potential workshop attendees. At the request of the SPC members, the sixth meeting included a demonstration of the pilot workshop by the research team. Through these developmental meetings, we designed a 60-min workshop including content on (1) firearms and firearm safety (2) how to talk to family members and friends (3) developing a plan for secure firearm storage.

Several themes emerged during developmental meetings (Table 1). SPC members indicated that the scope and frame of the workshop should focus on suicide prevention since that is the topic of trainings provided by SPCs. They also requested that the workshop include several modules that could be presented separately. Additionally, SPC members and the research team discussed strategies to identify and engage with local firearm experts to co-facilitate the workshop (e.g., contacting local gun shop owners, firearms advocacy groups, or tabling at firearm and suicide prevention events). SPC members also emphasized the importance of promoting the well-being of workshop attendees by acknowledging the difficulty of having these conversations and including resources for self-help. Finally, SPC members requested that the facilitator guide include information on specific populations (i.e., Veterans, working-aged men, sexual and gender minorities, women, those who carry service weapons, youth, other adults, Black Americans, Native Americans, and new firearm owners) that could be presented if members of the audience were from one of these groups.

#### Subject matter expert meetings

Three meetings were held with subject matter experts between December 2022 and February 2023 (total attendance rate = 91%). During these meetings, subject matter experts provided guidance on engaging multiple types of stakeholders and collaborated with the study team to develop and revise workshop content. They highlighted the need to clearly define the target audience of workshop (i.e., family members and loved ones of firearm owners) and to ensure that firearm secure storage and suicide prevention messaging aligned with the values of firearm owners and individuals from the suicide prevention community. One area of concern identified was being able to successfully promote the workshop to reach loved ones of firearm owners. Subject matter experts also emphasized the importance of discussing concerns about suicide and secure firearm storage with loved ones using direct, respectful, and sensitive language based on evidence-based practices. Finally subject matter experts helped refine workshop attendee assessment design and wording (i.e., survey) (Table 1).

#### Firearm expert meetings

Two members of the research team (GKK and HPC) met one-on-one with a total of seven firearm experts between January 2023 and April 2023. Meetings included a general overview of workshop content, and specifically focused on the information and messaging about firearms, safe handling, and secure storage, to assess feasibility and acceptability of workshop material. Analysis of firearm expert meetings revealed several themes.

Overall, firearm experts endorsed the value and need for community-based firearm suicide prevention efforts and viewed such efforts as in alignment with responsible firearm ownership. Firearm experts indicated there was hesitancy about discussing mental health topics among firearm owning individuals within their local communities and emphasized the importance of using credible, relatable messengers (e.g., gun shop owners, Veterans, firearm instructors) to deliver relevant information on firearms, including legislation, safe handling, and storage options. Firearm experts noted that firearm owners are often hesitant to seek mental health care or discuss their risk for suicide because of a fear of having their firearms involuntarily removed or losing the ability to own firearms. Finally, they emphasized the importance of explicitly stating that the workshop is focused on promoting collaborative, voluntary, and temporary limits in access to firearms during times of elevated risk of suicide.

#### Veteran family member meetings

Three family member/caregivers of Veterans at risk for suicide attended two team meetings and discussed strategies to identify potential workshop attendees, and also provided input on realistic conversations that family members might have with a loved one when discussing firearms and secure storage—these conversations were then incorporated into the workshop. Additionally, one team member (GKK) met separately over the course of two meetings with three family members who lost a loved one due to suicide by firearms to discuss suggested content for the workshop and characteristics of co-facilitators. When discussing the scope and frame of the workshop family members commented on the need for this type of workshop among family members of individuals at risk for suicide and expressed that they would have liked to have this workshop as a resource during their own struggles. Family members also discussed the importance of including a firearm expert co-facilitator, emphasizing voluntary limits in access to firearms during times of higher risk, and including resources for seeking mental healthcare.

### Pilot workshops

#### Content

The workshop includes three sections (1) firearms and firearm safety (about 25 min, delivered by firearm safety expert—parts can be delivered by SPC leader), (2) how to talk to family members and friends (about 35 min, delivered by SPC leader), and (3) developing a plan for secure firearm storage (about 10 min, delivered by SPC leader). Drawing from the evidence-based intervention, community reinforcement and family training (CRAFT), which was initially formulated for substance use and later extended to post traumatic stress disorder (PTSD), the workshop aims to equip participants with techniques to encourage their loved ones to seek treatment while protecting their own well-being (Roozen et al. 2010; Erbes et al. 2020; Croak et al. 2023). The research team adapted these skills for discussions about firearms in where the individuals may be hesitant or resistant to implementing secure storage measures.

#### Pilot workshop attendance

Pilot workshops were delivered in four NYS counties in-person (n = 3) or virtually (n = 1) between June and August 2023. Each workshop was presented by two co-facilitators: one firearms expert and one SPC leader. Facilitators recruited attendees by emailing list servs typically used by SPC members to recruit for events (e.g., caregivers at the VA, members of community suicide prevention groups) and using social media. An average of five individuals attended each workshop (range: 4–8), with the virtual workshop having the highest attendance (n = 8). Attendees (n = 23) were mainly white (95%) under fifty (69%) and about half women (47%; Table 2). The majority of participants (66%) had one or more family member or loved one serve in the military and four (17%) had formerly served in the military themselves.

**Table 2:** Demographics of pilot workshops and train-the-presenter attendees

Age group	Pilot workshop attendees (n = 23)	Train-the-presenter event (n = 28)
18–34	6 (26%)	9 (32%)
35–49	10 (43%)	14 (50%)
50–64	3 (13%)	1 (4%)
(4%)65+	4 (17%)	4 (14%)
*Gender*		
Woman	11 (48%)	14 (50%)
Man	6 (26%)	13 (46%)
Non-identifying	0 (0%)	1 (4%)
Did not answer	4 (17%)	0 (0%)
Did not collect	2 (1%)	0 (0%)
*Race/Ethnicity*^a^		
White	22 (95%)	25 (89%)
Black/African American	1 (4%)	0 (0%)
Hispanic/Latinx	1 (4%)	2 (7%)
*Have you served in the military?*		
Yes—Currently	0 (0%)	2 (7%)
Yes—Formerly	4 (17%)	10 (36%)
No	19 (83%)	16 (57%)
*Has your family member or loved one served in the military?*^a^		
Yes—Currently	5 (21%)	14 (50%)
Yes—Formerly	10 (43%)	21 (75%)
no	8 (34%)	4 (14%)

^a^Total add up to over 100% as attendees could select more than one option

#### Pilot workshop attendee feedback

Given the small sample size, we calculated only descriptive and not inferential statistics. Overall, pilot workshop attendees reported higher scores on the following items when reporting their perspectives prior to versus following workshop attendance: willingness and confidence to discuss secure firearm storage with a family member or loved one, openness to storing firearms more securely to prevent a suicide attempt, and endorsing that putting time and space between a firearm and an individual at risk for suicide can decrease risk (Table 3). Importantly, attendees endorsed high scores on these prompts when reporting their perspectives prior to attending the workshop. Themes for the open-ended question on how to improve workshop (n = 10) included increasing the length of the workshop above 60 min, using simpler language to relay information about firearms, and finding better ways to identify family and community members interested in attending the workshop.

**Table 3:** Pilot workshop attendee survey feedback

Statements	Before, M (SD)	After, M (SD)
Q1. Putting time and space between a firearm and an individual at risk for suicide can decrease risk	4.65 (0.65)	4.83 (0.39)
Q2. I would discuss secure firearm storage with family members or loved ones going through a hard time	4.22 (0.60)	4.83 (0.39)
Q3. I am confident in my ability to discuss secure firearm storage with a family member or loved one	4.00 (0.85)	4.65 (0.49)
Q4. I am open to storing firearms more securely to prevent a suicide attempt by a loved one, someone who lives with me, or myself	4.35 (0.65)	4.83 (0.39)
Q5. I would recommend this workshop to others	–	4.96 (0.37)

Questions were scored on a 5-point Likert scale (Strongly disagree = 1, Disagree = 2, Neither = 3, Agree = 4, Strongly agree = 5

#### Pilot workshop co-facilitator feedback

Every workshop co-facilitator agreed to complete the interview, for a total of eight interviews. We did not collect demographic data to ensure participant confidentiality and to enhance willingness and openness to discuss experiences facilitating the workshop. Overall, pilot workshop facilitators reported positive experiences collaborating with their co-facilitator and provided feedback on ways to revise each workshop component [Interviews present: n = 8 (100%) interviews] (Table 1). Some SPC co-facilitators noted initial difficulty connecting with firearm experts and indicated a desire for additional materials to facilitate engaging with firearm co-facilitators [n = 2 (25%)]. Firearm co-facilitators strongly emphasized the importance of using a credible messengers (e.g., gun shop owners, Veterans, firearm instructors) to deliver information relevant to firearms and firearm safety and indicated there is an interest within the firearm community to be part of firearm suicide prevention efforts and ensure the development of such efforts accurately reflect technicalities and values around responsible firearm ownership [n = 4 (50%)]. Firearm co-facilitators also lauded the workshop’s emphasis on voluntary, temporary, out-of-home firearm storage. SPC facilitators suggested meeting with their firearm co-facilitator to discuss comfortability and familiarity with workshop content (e.g., knowledge on NYS firearm related legislation, suicide prevention through secure firearm storage) and the process for co-facilitating the workshop [n = 4 (50%)]. The majority of co-facilitators recommended that the workshop be delivered in-person due to its sensitive content and to promote engagement, but were also open to holding virtual meetings [n = 7 (87.5%)]. SPC co-facilitators noted that many workshop attendees were providers, perhaps because providers are the typical audience for SPC events, and expressed the desire to partner more extensively with other community groups (e.g., firearm owning groups) to better identify family members and loved ones of individuals at risk for suicide to attend the workshop [n = 4 (50%)]. Following a team review of interview data, the length of workshop increased from 60 to 75–90 min and the workshop slides and handouts were consolidated. The research team also revised the facilitator’s guide to include a summary sheet for co-facilitators (e.g., one for SPC members and one for firearms experts) and a one-page handout for SPC members to use in their recruitment of firearm experts.

### Train-the-presenter event attendance

In October 2023, a 1-day training event for was held at the D’Aniellio Institute for Veteran and military Families (IVMF) in Syracuse, New York. A total of 42 individuals attended the train-the-presenter event, representing 26 New York State counties; 30 attended in-person and 12 attended virtually.

### Train-the-presenter event feedback

At the event, 38 attendees completed a short planning survey and 28 completed an anonymous demographic and feedback survey. Attendees were mainly white (89%) under fifty (82%), and half were women (50%). The majority of participants (86%) had one or more family member or loved one serve in the military and almost half (42%) served or currently serve in the military (Table 2). Most attendees were experts in suicide prevention (n = 25; 64%), about one-third (n = 9; 32%) reported being able to serve as an expert in both firearms and suicide prevention, and about 11% (n = 4) of attendees identified as firearm experts. Overall, attendees reported higher scores in comfortability talking about firearm safety for suicide prevention following the training event and endorsed the training presentation and activities (Table 4).

**Table 4:** Train-the-presenter confidential feedback

Statements	M (SD)
Q1. Prior to today’s training, I felt comfortable talking about firearm safety for suicide prevention	4.07 (0.65)
Q2. Following today’s training, I felt comfortable talking about firearm safety for suicide prevention	4.54 (0.57)
Q3. The presentation portions of the training were effective	4.29 (0.45)
Q4. The interactive activities of the training were effective	4.32 (0.54)
Q5. I would recommend this training to others	4.64 (0.48)
Open ended: Please describe any topics or issues you hoped to discuss today that were not included in the training:	Some participants suggested incorporating more behavioral health aspects and focusing less on the technical details (“nuts and bolts”) of firearms
	There was a request for incorporating additional phrases or language to facilitate difficult conversations within the training
	There was a request to incorporate testimonials from those with lived experience who have utilized the training with loved ones

Questions were scored on a 5-point Likert scale (Strongly disagree = 1, Disagree = 2, Neither = 3, Agree = 4, Strongly agree = 5

Twenty-six attendees (70%) from 15 counties reported an intent to facilitate a workshop by the end of 2024. Themes (n = 5) for the open-ended questions included facilitators wanting more information about suicide prevention and behavioral health, guidance on language to have a conversation about secure firearm storage and suicide prevention, as well as a desire to incorporate testimonials from people who have taken the training (Table 4).

## Discussion

We aimed to develop a community-based workshop to empower family members and loved ones to discuss secure firearm storage for suicide prevention. We found that by working with SPC leaders, local firearm experts, subject matter experts, and family members of individuals at risk for suicide, we were able to develop, refine, and disseminate a workshop on a sensitive topic that was considered acceptable by all stakeholder groups. Pilot workshop attendees reported positive experiences and suggested that the workshop enhanced their willingness to and confidence in discussing firearm storage with their loved ones and pilot workshop facilitators overall reported positive experiences delivering the workshop. Although this workshop was developed around the needs of a specific community, we see some lessons as generalizable to the larger field of lethal means education.

Our findings complement the findings of other similar studies showing that it is possible to engage and build relationships within the firearms community through participatory action research and that there are members of the firearms community interested in promoting suicide prevention (Constans et al. 2023; Betz et al. 2022). This was seen both in the strong enthusiastic response of our firearm expert stakeholders in the initial workshop development process and in the strong working relationships developed between co-facilitators across the four pilot communities. In many cases, the co-presenters continued discussing programming after their pilot workshop. Given the prior research indicating that military veterans, law enforcement, and firearm instructors and retailers are perceived as trustworthy messengers by both firearm owners and non-firearm owners, the strong response would suggest that it is feasible to identify individuals willing to serve as these messengers (Pallin et al. 2019; Bond et al. 2022; Anestis et al. 2021). The firearm co-facilitators’ responses in their post-treatment interviews underscored that they believed in the importance of this role, particularly when emphasizing the importance of collaborative decision making for voluntary, temporary, out-of-home firearm storage.

The piloting phase also revealed a potential challenge for community-based lethal means education. Although our workshop was well-received by those who attended, the reach was limited both in the overall number of attendees at these pilot workshops, and the facilitators sharing in their post-workshop interviews that a high proportion of attendees were already trained mental health professionals. This challenge reaching non-professional audiences (i.e., “lay audiences”) is not unique to this effort. In a larger scale rollout of the Conversations on Access to Lethal Means—General Public program, over 68% of attendees were mental health professionals (Ellison et al. 2023). Facilitators emphasized the importance of thinking through promotional efforts to reach family members and loved ones of firearm owners and individuals at risk for suicide in the community. Given that SPCs often focus on mental health professional audiences, it would be sensible to expect that their default promotional strategies would be tailored to those audiences. Since the vast majority of lethal means safety education focuses on medical providers who can be incentivized to attend through continuing education credits and the majority of gatekeeper trainings target the employing hospitals or school systems, there are few models for how to engage the general public in workshop-based suicide prevention education (Rosen et al. 2022; Prater et al. 2021; Quinnett 2023; Teo et al. 2022). It is likely that further implementation-focused research in the lethal means safety space is needed to identify the best strategies for reaching lay populations.

For those who did attend the workshop, we were pleased to learn that the dual-presentation strategy was well-received. This study adds to the evidence base that community-involved trainings programs are a promising and acceptable approach to firearm suicide prevention among community members (Ellison et al. 2023). It is important to note that the impacts of such trainings on actual storage behaviors were not evaluated in this trial. Since a large number of attendees were mental healthcare professionals, we are cautiously optimistic that they would show the same benefits observed in clinician oriented research, which suggests that participation in lethal means safety workshops is associated with increased number of lethal means safety conversations with clients (Sale et al. 2018). While family members do not have access to the same number of at-risk individuals as mental health counselors or emergency room doctors, they have the advantage of sustained, repeated contact with a smaller number of firearm owners, which provide multiple opportunities to revisit secure firearm storage conversations. Furthermore, even if these conversations are less than optimal, family members are up to twice as likely to be aware of suicide warning signs as healthcare professionals, meaning that some may have these crucial discussions with at-risk individuals that healthcare professionals will never reach. Therefore, we are hopeful that across both clinician attendees and lay audiences, that these increased conversations will be associated with greater adoption of secure storage practices by firearm owners (Albright and Burge 2003; Barkin et al. 2008). Future research should assess whether workshop attendees have more conversations around secure storage with their loved ones and in turn assess whether those conversations are associated with secure storage practices in the household.

### Strengths and limitations

The main strength of this study was the willingness and enthusiasm of multiple stakeholder groups to participate in the development of the workshop, and the enduring and collaborative relationships among co-facilitators that we observed. Another novel aspect of this study was the model of including co-facilitators with expertise in both suicide prevention and firearms. This structure allowed for shared messaging and collaboration among facilitators while allowing each facilitator to present on their area of expertise.

Limitations during the development phase included a focus on veteran family members. While some SPC members and our firearm experts represented other firearm owning groups (e.g., law enforcement, hunters), selecting our family perspectives from one specific firearm owning sub-population (veterans) might limit the generalizability of the content to other audiences. Limitations during the evaluation phase included the lack of racial/ethnic diversity among pilot workshop attendees, the small number of attendees at the pilot workshops, and the limited attendance of family members and loved ones of firearms owners and individuals at risk for suicide versus providers. While attendance at in-person events was lower and may be more challenging for some family members, co-facilitators noted the importance of offering both in person and virtual options to meet the preferences of different community members.

### Future directions

The research team was awarded an additional grant from the New York Health Foundation to continue to support the implementation and dissemination of the workshop across New York State. The funds will address the low reach and diversity of the pilot workshops by supporting “on-the-ground” recruitment partnerships with outreach coordinators in select communities. Additionally, we will focus on expanding the diversity of workshop attendees by working with community-based veteran organizations, faith communities, and firearm advocacy organizations.

## Conclusions

The current study used a multistakeholder engagement framework to develop a workshop on firearm safety for suicide prevention that will be disseminated throughout New York State. These findings suggest the feasibility of this approach and the potential for community-based workshops to disseminate information on this important topic.

### Supplementary Information

Supplementary Material 1.

Supplementary Material 2.

Supplementary Material 3.

Supplementary Material 4.

Supplementary Material 5.

## Data Availability

The datasets used and/or analyzed during the current study are available from the corresponding author (HC) upon reasonable request.

## Abbreviations

PCORI: Patient-Centered Outcomes Research Institute
SPC: Suicide prevention coalition
CRAFT: Community reinforcement and family training
PTSD: Post-traumatic stress disorder
