# Supplementary material for: Engaging suicide prevention and firearm stakeholders in developing a workshop promoting secure firearm storage for suicide prevention
Source: Inj Epidemiol. 2024 Jun 14;11:26. doi: 10.1186/s40621-024-00511-7 (PMC11179275; doi:10.1186/s40621-024-00511-7)

Firearms and suicide

The most common method of suicide in the U.S. — **53%**

Most firearm fatalities are from suicide in the U.S. — **60%**

Suicide attempts with firearms are fatal — **90%**

Once you pull the trigger, you can't change your mind or be rescued

Suicidal crises are short – often only a few minutes long. To prevent suicide, we need to get people through these intense periods alive

Suicidal crises are time limited

Putting **time** and **space** between a person at risk for suicide and a firearm can save a life

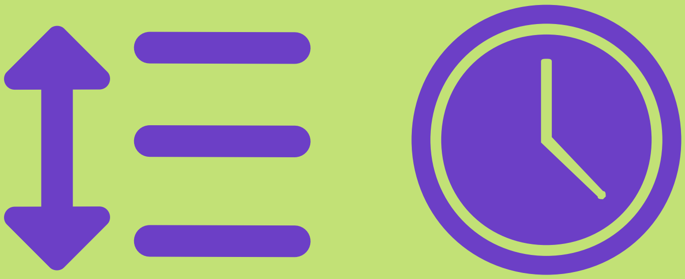

Supplement: Supplementary file 1 — Supplementary Material 1. [file 40621_2024_511_MOESM1_ESM.pdf]
