# Supplementary material for: Engaging suicide prevention and firearm stakeholders in developing a workshop promoting secure firearm storage for suicide prevention
Source: Inj Epidemiol. 2024 Jun 14;11:26. doi: 10.1186/s40621-024-00511-7 (PMC11179275; doi:10.1186/s40621-024-00511-7)
Supplement: Supplementary file 2 — Supplementary Material 2. [file 40621_2024_511_MOESM2_ESM.pdf]

<u>Having an Effective Conversation (O.P.T.I.O.N.)</u>

Prepare <u>OPTIONS</u> that limit access or add time/distance:

- Review ***Gun Storage for your Lifestyle*** for 2-3 choices that make sense
- Research the details. For example: Cost, Phone #s, and how it fits in your home

Bring the right <u>PEOPLE</u> to the table. Groups are overwhelming, you just need:

- Someone your loved one can discuss personal firearm storage details with
- Someone who can disagree respectfully with your loved one
- Someone who knows enough about firearms for a productive discussion

If you can do all three, then a one-on-one conversation might work best!

Pick the right <u>TIME</u>:

- When you are both calm and don't feel rushed
- When there are few distractions and you are thinking clearly

Speak from the "<u>I</u>" but also provide relevant <u>INFORMATION:</u>

- "The I:" Share YOUR concerns about suicide
  - What warning signs have you noticed?
  - Make it clear when you're sharing opinions with "I feel," "I think," etc.
- Limit facts and stories
  - What facts stand out to you? Why does it matter?
  - What facts speak to your loved one's values?
- Stay focused on recent incidents and concerns
  - Don't bring up the distant past
  - Talk about worries (and hopes!) for the future

Be <u>OPEN</u> to your loved one's perspective:

- Don't lecture. Speak briefly to give your loved one space to respond.
- Pay attention to their reactions, try to understand them
- Understand their values:
  - Why is firearm ownership important to them?
  - What do they like and value about the current storage plan?

Be prepared for a "<u>NO</u>," (and remember that's okay!):

- Remind yourself that by speaking from "<u>the I</u>," they now understand you better.
  - Let them think about it, and then come back at a later <u>TIME</u>
- By being <u>OPEN</u> you now learned about why some suggestions might not fit
  - Use that knowledge to research new <u>OPTIONS</u> that might work better

O.P.T.I.O.N. Worksheet

OPTIONS for Storage:

____
____
____
____
____

PEOPLE I might want to involve:

Can my loved one…	With Me?	IF NO: Then who can I ask to help?
…discuss their storage details		
…disagree calmly		
…trust firearm advice		

TIME where WE will be most effective:

____
____

INFORMATION and concerns that I want my loved one to know:

____
____
____
____
____
____

Tips to stay OPEN to your loved one's perspective:

- Speak briefly to give your loved one space, **LISTEN** to understand your loved one's perspective. It will inform whether to respond and how to respond.

- Questions to better understand their perspective:
  - Why is firearm ownership important to you? ____________________________
    ____
  - What works about our current storage plan? ____________________________
    ____

Things you can do if they say "NO":

____
____
____
