# Supplementary material for: Engaging suicide prevention and firearm stakeholders in developing a workshop promoting secure firearm storage for suicide prevention
Source: Inj Epidemiol. 2024 Jun 14;11:26. doi: 10.1186/s40621-024-00511-7 (PMC11179275; doi:10.1186/s40621-024-00511-7)
Supplement: Supplementary file 3 — Supplementary Material 3. [file 40621_2024_511_MOESM3_ESM.pdf]

Confidential Survey

Please complete this brief survey to provide feedback on today's training. Your responses are completely confidential and will not be linked to your name or any other information. Please feel free to skip any items that you do not feel comfortable answering. Please contact Dr. Gabriela Khazanov (kattang@sas.upenn.edu) if you have any questions or feedback.

1. **Prior to today's training**, I felt comfortable talking about firearm safety for suicide prevention	Strongly Disagree	Disagree	Neither	Agree	Strongly Agree
2. **Following today's training**, I feel comfortable talking about firearm safety for suicide prevention	Strongly Disagree	Disagree	Neither	Agree	Strongly Agree
3. The presentation portions of the training (Powerpoint, demonstrations) were effective	Strongly Disagree	Disagree	Neither	Agree	Strongly Agree
4. The interactive activities of the training (e.g., practice, breakouts) were effective	Strongly Disagree	Disagree	Neither	Agree	Strongly Agree
5. I would recommend this training to others	Strongly Disagree	Disagree	Neither	Agree	Strongly Agree

8. Please describe any topics or issues you hoped to discuss today that were not included in the training:

____

____

____

Demographic Information:

9. Age group:

O 18-34
O 35-49
O 50-64
O 65+

10. Gender:

O Female
O Male
O Other __________

11. Have you served in the military?

O YES - Currently
O YES - Formerly
O NO

12. Do you have a family member or loved one who has served in the military?

O YES - Currently
O YES - Formerly
O NO

13. Race/Ethnicity (*check all that apply*):

□ White
□ Asian
□ Hispanic/Latinx
□ Other _________________
□ Black/African American
□ American Indian/Alaskan Native
□ Native Hawaiian/Pacific Islander

Plans to Conduct a Firearm Safety for Suicide Prevention Workshop (Not Confidential)

We really value your input! Thank you for completing the survey.

Name:________________ **Organization:**________________

1. I consider myself an expert in:
   O Suicide prevention
   O Firearms
   O BOTH suicide prevention & firearms

2. At this time, I plan on facilitating a Firearm Safety for Suicide Prevention workshop in my community

   O YES, by the end of 2024
   O YES, but in 2025 or later
   O NO
   O NOT SURE (please describe: ________________________________)

3. I am confident I will be able to facilitate a Firearm Safety for Suicide Prevention workshop in my community	Strongly Disagree	Disagree	Neither	Agree	Strongly Agree
4. My community will want to participate in a Firearm Safety for Suicide Prevention workshop	Strongly Disagree	Disagree	Neither	Agree	Strongly Agree

5. Please describe potential barriers to facilitating a Firearm Safety for Suicide Prevention workshop in your community:

____

____

6. Please describe anything you think would be helpful to support you in facilitating a Firearm Safety for Suicide Prevention workshop in your community:

____

____

7. Are you interested in working with us to promote and increase attendance at your workshop?

   O YES (*Please provide an up-to-date e-mail address:* ___________________)
   O NO

8. To understand how you already recruit attendees for your workshops, please check which of the following groups you or your coalition already have a working relationship with:

   □ Mental health advocacy groups (e.g., NAMI [National Alliance on Mental Illness])
   □ Veterans' groups (e.g., VFW [Veterans of Foreign Wars])
   □ Religious/faith groups
   □ Firearm advocacy organizations (e.g., NSSF [National Shooting Sports Foundation])
   □ Networks of health and/or mental health providers or clinicians
   □ Other ___________________________________
