# Supplementary material for: Engaging suicide prevention and firearm stakeholders in developing a workshop promoting secure firearm storage for suicide prevention
Source: Inj Epidemiol. 2024 Jun 14;11:26. doi: 10.1186/s40621-024-00511-7 (PMC11179275; doi:10.1186/s40621-024-00511-7)
Supplement: Supplementary file 4 — Supplementary Material 4. [file 40621_2024_511_MOESM4_ESM.pdf]

Please complete this brief survey to provide feedback on today's workshop. Your responses will be used to understand your experience of the workshop and improve it for future participants. Your responses are completely confidential and will not be linked to your name or any other information. Additionally, the workshop facilitator will not see your responses. Please feel free to skip any items that you do not feel comfortable answering. As a token of appreciation for completing the survey, you will receive a small gift. Please contact Dr. Gabriela Khazanov (kattang@sas.upenn.edu) if you have any questions or feedback.

	**Before the workshop, I thought…**					**Right now, I think…**				
Putting time and space between a firearm and an individual at risk for suicide can decrease risk	Strongly Disagree	Disagree	Neither	Agree	Strongly Agree	Strongly Disagree	Disagree	Neither	Agree	Strongly Agree
I would discuss safe firearm storage with family members or loved ones going through a hard time	Strongly Disagree	Disagree	Neither	Agree	Strongly Agree	Strongly Disagree	Disagree	Neither	Agree	Strongly Agree
I am confident in my ability to discuss safe firearm storage with a family member or loved one	Strongly Disagree	Disagree	Neither	Agree	Strongly Agree	Strongly Disagree	Disagree	Neither	Agree	Strongly Agree
I am open to storing firearms more securely to prevent a suicide attempt by a loved one, someone who lives with me, or myself	Strongly Disagree	Disagree	Neither	Agree	Strongly Agree	Strongly Disagree	Disagree	Neither	Agree	Strongly Agree
I would recommend this workshop to others.	________________					Strongly Disagree	Disagree	Neither	Agree	Strongly Agree

Please provide feedback on the workshop to help us improve it:

____

____

Demographic Information:

Age group: ◻ 18-34 ◻ 35-49 ◻ 50-64 ◻ 65+ **Gender**: ◻ Female ◻ Male ◻ Other ________

Race/Ethnicity (check all that apply): ◻ White ◻ Black/African American ◻ Asian ◻ American Indian/Alaskan Native ◻ Native Hawaiian/Pacific Islander ◻ Hispanic/Latinx ◻ Multiracial ◻ Other ________

Have you served in the military? ◻ Yes – Currently ◻ Yes – Formerly ◻ No

Has your family member or loved one served in the military? ◻ Yes – Currently ◻ Yes – Formerly ◻ No

We really value your input! Thank you for completing the survey.
