# Supplementary material for: Engaging suicide prevention and firearm stakeholders in developing a workshop promoting secure firearm storage for suicide prevention
Source: Inj Epidemiol. 2024 Jun 14;11:26. doi: 10.1186/s40621-024-00511-7 (PMC11179275; doi:10.1186/s40621-024-00511-7)
Supplement: Supplementary file 5 — Supplementary Material 5. [file 40621_2024_511_MOESM5_ESM.pdf]

Participant ID#:__________ Interviewer Initials: __________ Date: ____________

Coder:____________ Date of workshop:____________

Stakeholder Group (*insert 'X' to indicate*)

**Suicide Prevention Coalition Member _______**	**Firearm Expert _________**

Workshop Facilitator Interview Guide

Introduction:

Hello, thank you so much for agreeing to talk to us. Today we are going to discuss your experiences with facilitating the workshop "Firearm Safety for Suicide Prevention".

I'd like to remind you that any information you share today is confidential. Your comments will not be linked to any personally identifying information. When sharing results, we will do so in aggregate so that individual comments cannot be traced back to you. There are no right or wrong answers to any questions – we are just interested in hearing your thoughts and perspectives. The interview will take 30-40 minutes.

Are you in a place private place where you feel comfortable conducting this interview? (If no, wait until they have changed locations or reschedule the interview for another time.)

Do you have any questions before we begin?

Rapid Qualitative Coding

(Instructions: fill out responses to probes in corresponding 'Notes' section during interview)

Domain	Probes	Notes
**Introduction**	1. Can you describe your previous experience with suicide prevention and firearm safety?<br>a. How many years have you been in this field?<br>2. What other trainings related to lethal means or firearms have you offered to the community?	1.<br>*1.a.*<br>2.
**Recruitment**	1. How did you promote attendance at the workshop?<br>a. What specific groups did you work with or contact?<br>2. Who were attendees in terms of background, roles/jobs, and demographics (gender, race/ethnicity, age)?<br>3. Do you have suggestions for promoting attendance that we can share with other groups?	1.<br>*1.a.*<br>2.<br>3.
**Workshop Delivery**	1. Can you describe your experience facilitating the workshop?<br>a. What went well? What didn't go so well?	1.<br>*1.a.*

	b. What were the most frequent topics of discussion?<br>c. Did any topics come up that were not covered in the workshop?<br>2. How did the workshop format (in-person, virtual, or hybrid) impact your experience?<br>3. What interactive components, if any, did you use in the workshop?	*1.b*<br>*1.c.*<br>2.<br>3.
**Co-Facilitator**	1. Who was your co-facilitator?<br>a. How did you find them?<br>2. What was your experience like working with them?<br>a. How many times did you meet prior to putting on the workshop?<br>b. Were there any barriers to working together effectively?<br>c. How did you or the workshop benefit from working with them?	1.<br>2.<br>*2.a.*<br>*2.b.*<br>*2.c.*
**Facilitator guide and materials**	1. What training materials did you use (facilitator guide, short Tim video, long Tim/Katie video)?<br>2. Would you suggest any changes to the materials?	1.<br>2.
**Sustainment**	1. Do you plan on delivering this workshop again?<br>a. What opportunities in your community may help you continue to facilitate this workshop?<br>b. What barriers do you anticipate to continuing to facilitate this workshop?<br>2. Did you learn anything from delivering the workshop that will be useful to you in your general role/job moving forward?	1.<br>*1.a*<br>*1.b*<br>2.
**Other**	1. What suggestions do you have for other groups thinking about facilitating this workshop?<br>2. Is there anything else you think is important for us to know?	1.<br>2.

Logistics Matrix

(Instructions: fill out below table during interview, information not gathered during interview, ask explicitly at end)

Workshop Delivery			
Attendees	<10 people	10-19 people	>20 people (How many?)
Delivery modality	In-person	Virtual	Hybrid
Timing	~60 min	60-90 minutes	>90 min
What training supports did you use?	Facilitator guide	Short Tim video	Long Tim/Katie video
Makeup of the audience (select all that apply)	Family members/loved ones of individuals at risk for suicide	Family members/loved ones of firearm owners	Individuals at risk for suicide
	Individuals who own firearms	Special populations (circle if facilitator mentions any of these populations): Veterans, working-aged men, women, Black Americans, Native Americans, sexual and gender minorities, youth, older adults, those who carry service weapons, and new firearm owners	
	Clinicians/providers:	Coalition leaders/community organizers	Other:

Process notes (observations about openness etc., themes that arose):
